# Effects of dietary metabolizable energy and crude protein levels on laying performance, egg quality and fecal microbiota of Taihe Silky Fowl during the peak laying period

**DOI:** 10.5713/ab.24.0446

**Published:** 2024-10-25

**Authors:** Wentao Li, Lixia Kai, Yutian Shen, Weifa Su, Yuqing Fan, Yizhen Wang, Zeqing Lu

**Affiliations:** 1 National Engineering Research Centre for Green Feed and Healthy Farming, Zhejiang University, Hangzhou 310058, China; 2 Key Laboratory of Animal Nutrition and Feed Nutrition of Zhejiang Province, Zhejiang University, Hangzhou 310058, China; 3 Institute of Feed Science, College of Animal Science, Zhejiang University, Hangzhou 310058, China; 4 Taihe Silky Fowl Industrial Technology Joint Research Center of Zhejiang University, Zhejiang University, Hangzhou 310058, China; 5 Taihe Silky Fowl Industry Development Center of Taihe County, Ji’an 343700, China

**Keywords:** Crude Protein, Fecal Microbiota, Laying Performance, Metabolizable Energy, Nutritional Requirement

## Abstract

**Objective:**

The experiment aimed to study the effect of dietary metabolizable energy (ME) and crude protein (CP) on laying performance, egg quality, serum routine biochemical and lipid metabolism indicators, the apparent digestibility of nutrients, and fecal microbiota of Taihe Silky Fowl (TSF) during the peak laying period.

**Methods:**

A total of 540 26-week-old TSF female fowls were randomly allocated to 9 groups with 5 replicates per group and 12 fowls per replicate. The fowls were fed with a 3×3 factorial arrangement of treatments diets (ME:10.88,11.30, or 11.72 MJ/kg; CP: 15, 16, or 17%).

**Results:**

With the increasing CP level, the egg weight (p = 0.023), egg production (p = 0.047), and egg mass (p = 0.022) enhanced, while the feed conversion rate (FCR) (p = 0.023) decreased. As the ME levels grew, the average daily feed intake (p<0.001) and FCR (p = 0.045) decreased. With enhanced ME, the triglycerides (p = 0.037), total cholesterol (p = 0.041), and high-density lipoprotein cholesterol (p = 0.028) increased, whereas the low-density serum lipoprotein cholesterol (p = 0.039) decreased. The apparent digestibility of CP increased as the ME level increased (p = 0.029) and as the CP level decreased (p = 0.027). At the same time, the apparent digestibility of gross energy increased as the ME level increased (p = 0.018). Different levels of ME or CP changed the composition of fecal microbiota, 17% CP increased the abundance of *Bifidobacterium*.

**Conclusion:**

It is suggested that 10.88 MJ/kg dietary ME and 17% CP level are suitable for the nutritional requirements of TSF during the peak laying period.

## INTRODUCTION

**Figure 1:**
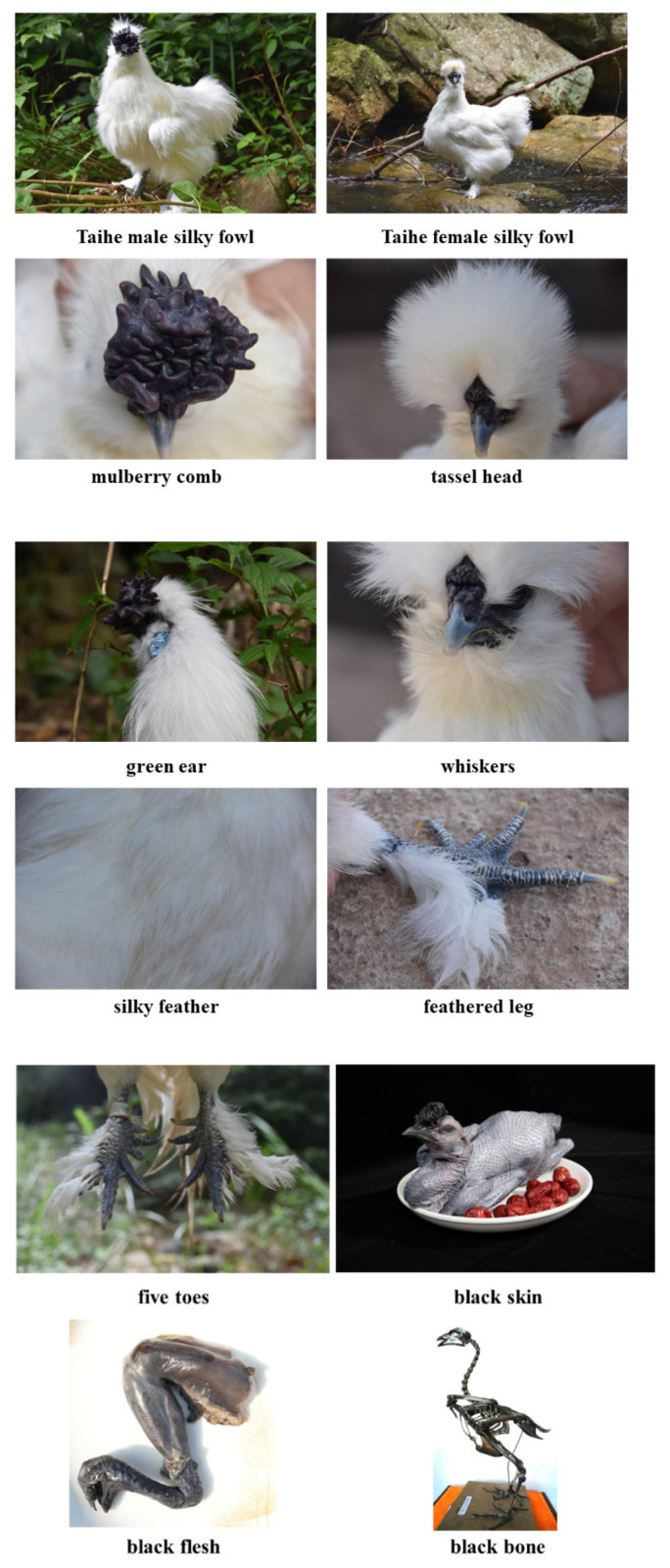
The “ten perfect” appearance characteristics of TSF. TSF, Taihe Silky Fowl.

Taihe Silky Fowl (TSF, Gallus *gallus domesticus* Brisson), native to Taihe County, Ji’an City, China, is an international standard breed with a history of more than 2,000 years [1]. As depicted in Figure 1, it has a delicate and exquisite body, with ten characteristics of “mulberry comb, tassel head, green ear, beard, silky feather, feathered leg, five toes, black skin, black flesh, black bone”, making it become a precious poultry breed in China [2].

TSF is a dual-purpose chicken for meat and egg production (EP). As for EP, female fowl exhibit a low laying rate and strong nesting nature, with an annual 80 to 100 eggs [3]. The reasons for this low EP may be that the breed has not undergone long-term genetic selection, and no clear nutritional requirements have been determined for the laying period, especially during the peak laying period. Hence, studies are necessary on the suitable nutritional requirements and practical levels of nutrients in diets for TSF, especially metabolizable energy (ME) and crude protein (CP). Dietary ME and CP, the main contributors to feed cost, are two major nutritional variables for evaluating the feed nutritive value. In the face of rising feed prices, many farmers are increasingly concerned about the feed conversion ratio (FCR) and economic returns. Meanwhile, ME and CP levels are important factors affecting laying performance: reasonable levels can improve EP and reduce FCR. Gunawardana et al [4] found that a 14.89% to 17.38% increase in the CP level, an EP increase of 6.5%, and an increase of 2.38 g average egg weight (EW) in molted Hy-Line W-36 hens. Li et al [5] reported that with the gradual increase in the ME level (2,400, 2,550, 2,700, and 2,850 kcal/kg), the FCR decreased significantly in Lohmann Brown laying hens from 26 weeks to 38 weeks.

Many studies have evaluated the effect of dietary ME and CP on commercial layers, while little information is available on TSF, especially during the peak laying period. Therefore, this study aimed to determine the effect of dietary ME and CP on TSF laying performance, egg quality, serum routine biochemical and lipid metabolism indicators, apparent digestibility of nutrients, and fecal microbiota of TSF at 27 to 37 weeks of age. The results are expected to provide an important nutritional reference for the efficient rearing of this precious dual-purpose fowl breed rearing.

## MATERIALS AND METHODS

### Animal care

The experiment was conducted following the Chinese guidelines for animal welfare and approved by the Animal Welfare Committee of the College of Animal Sciences, Zhejiang University (ZJU20170466).

### Animals, diets and experimental design

In this experiment, the TSF were reared at the premises of Xichangfengxiang Poultry Industry Co., Ltd. (Ji’an, China). A total of 540 26-week-old female TSF, were randomly allocated to 9 groups with 5 replicates per group and 12 birds per replicate (one replicate in three cages, 4 birds per cage [45×45×50 cm]) in A-type conventional cages arranged in a brick and tile house with windows. During the pre-feeding period (one week, 11.30 MJ/kg ME and 16% CP, mash feed), treatments were assigned based on the mean EP. Subsequently, the layers were arranged in a 3×3 factorial arrangement of treatments, which included 3 ME levels (10.88,11.30, or 11.72 MJ/kg [also 2600, 2700, or 2800 kcal/kg ME]) and 3 CP levels (15, 16, or 17% CP) at 27-week-old. The settings of ME and CP levels were referenced to the recommended levels in “Technical Regulations for the Production of Commercially-Available Taihe Silky Fowl Breeders” (DB36/T 667-2018, Fan et al [6]) and Commercial Breeder Chicken Feed of Ji’an Aonong Development Co., Ltd. (Ji’an, China). The experimental period lasted for 11 weeks. The composition and nutrient levels of diets were shown in Table 1. Cages were located in a ventilated area with a temperature between 18°C to 27°C, relative humidity between 60% to 70%, and 16 h/d of illumination (10 to 20 lx). Diets in mash form and water were offered *ad libitum* throughout the entire experimental period.

### Laying performance

During the experimental period, feed intake was calculated weekly, and EP, EW, broken EP, and mortality (low and not insignificant values were not displayed) were recorded daily. The feed consumption was adjusted based on mortality each day. Based on these data, the average daily feed intake (ADFI), egg mass (EM, EM = EP×EW), and FCR (FCR = ADFI/EM) were calculated.

### Egg quality

At the end of the experiment, due to the decrease in EP of laying hens, 6 eggs could not be collected from some replicates. Therefore, we adopted a method of collecting 30 eggs per group as the detection standard (n = 30) to assess the egg quality parameters. Albumen height, Haugh unit, yolk color, and eggshell strength were measured using an egg quality multitester (DET-6000; Nabel, Kyoto, Japan). Specifically, eggshell thickness was measured at three sites (air cell, equator, and sharp end) using a spiral micrometer (JDB03; SanLiang, Dongguan, China) and the mean value was calculated. The weight of the eggshell after separation from the egg was measured using an analytical balance (XPE204; Mettler Toledo, Columbus, OH, USA).

### Blood and fecal samples

Three days before the end of the experiment, 2 healthy fowls were randomly selected from each replicate and housed individually in a single cage at 10 birds per treatment. Feces were collected twice a day at 8:00 am and 4:00 pm for 3 days. The fowls were fasted for 12 hours before feces collection and provided *ad libitum* access to water. The fecal sample collection and determination method of the apparent digestibility of nutrients were performed according to Zhang [7]. To give a brief description, the feed intake and fresh feces weight were recorded after removing the white uric acid of the hens. In the metabolic test, the feathers and other debris were removed from the excreta, samples were homogenized by treatment, added with H2SO4 (10%) to 3% weight of feces for nitrogen fixation, and frozen for storage. Specifically, gross energy measurement was conducted using a Parr adiabatic oxygen bomb calorimeter (Parr 6400, Parr Instrument Co., Moline, IL, USA). On the last day, fresh rectal swab feces were collected in sterile lyophilized tubes for microbial 16s rRNA gene analysis. Subsequently, blood samples were collected by wing venipuncture, centrifuged at 3,500×g for 10 min at 4°C, and stored at −20°C for later analysis. The serum routine biochemical indicators included alanine aminotransferase (ALT), aspartate aminotransferase (AST), total protein (TP), albumin (ALB), globulin (GLOB), albumin/globulin (A/G) and the serum lipid metabolism indicators included triglycerides (TG), total cholesterol (TCHO), low-density lipoprotein cholesterol (LDL-C), high-density lipoprotein cholesterol (HDL-C), uric acid were determined by an automatic biochemical analyzer (UniCel DxC 600 Synchron; Beckman Coulter, Brea, CA, USA).

### DNA extraction and microbial 16S rRNA gene sequencing analysis

A genomic DNA extraction kit was utilized to extract the microbial DNA from feces (Sangon Biotech, Shanghai, China) according to the manufacturer’s protocal. The DNA was quantified using the NanoDrop2000 system (Thermo Scientific, Wilmington, NC, USA), and its quality was assessed by 1% agarose gel electrophoresis. The V4 hypervariable regions of the bacterial 16S rRNA gene were amplified using the specific primers 515F (5′-GTGCCAGCMGCCGCGG-3′) and 806R (5′-GGACTACHVGGGTWTCTAAT-3′). Subsequently, those genes were sequenced on an Illumina MiSeq platform (Illumina, Inc., San Diego, CA, USA) and and the raw sequencing data were processed using Quantitative Insights into Microbial Ecology 2 (QIIME2) software. Alpha diversity (Shannon index) was calculated by Mothur (v1.30.2; http://mothur.org/wiki/calculators/). Clusters were based on similarity in abundance between bacterial genera or samples and the heatmap was visualized using the package pheatmap (1.0.8) in R software (version 3.3.1). Linear discriminant analysis (LDA) coupled with linear discriminant analysis effect size (LEfSe, http://huttenhower.sph.harvard.edu/galaxy/root?tool_id=lefse_upload) was used to identify differentially abundant taxa at the genus level among various groups.

### Statistical analysis

Statistical analyses were conducted using SPSS 27.0 Software (SPSS Inc., Chicago, IL, USA). In this study, dietary ME and CP levels were analyzed using a general linear model to evaluate main effect and interaction effect, particularly, Kruskal-Wallis H test to analyze the fecal microbiota. p<0.05 was regarded as statistically significant, and 0.05<p<0.10 considered to indicate a trend. Data were presented as mean & standard error of mean.

## RESULTS

### Laying performance

As shown in Table 2, compared with the 15% CP diet, the EW, EP and EM increased (p<0.05) in the 17% CP diet and the FCR decreased (p<0.05) in the 16% and 17% CP diets. Specifically, the EP was 3.78% higher in the layers of fed diet containing 17% CP compared with the 15% CP diet. Compared with the 16% CP diet, the EW and EM were increased (p<0.05) in the 17% CP diet. In terms of ME, with its level increasing, the ADFI and FCR decreased (p<0.05).

### Egg quality

As can be seen in Table 3, dietary ME or CP levels had no main or interaction effects on albumen height, Haugh unit, yolk color, eggshell weight, eggshell thickness, or eggshell strength (p>0.05).

### Serum routine biochemical and lipid metabolism indicators

As can be seen in Table 4, dietary ME or CP levels had no main effects on the serum concentrations of ALT, AST, ALB, and A/G (p>0.05), while TP and GLOB exhibited an increased trend with the increase in CP level (0.05<p<0.10). Table 5 reflects the serum lipid metabolism. Compared with the 10.88 MJ/kg ME diet, the concentration of TG, TCHO and HDL-C were increased (p<0.05), and the LDL-C was decreased (p<0.05) in 11.72 MJ/kg ME diet.

### Apparent digestibility of nutrients

As demonstrated in Table 6, compared with the 15% CP diet, the apparent digestibility of CP was decreased in the 17% CP diet (p<0.05). Compared with the 10.88MJ/kg ME diet, the apparent digestibility of CP and gross energy were increased in the 11.72 MJ/kg ME diet (p< 0.05). The apparent digestibility of CP (p = 0.029) increased as the ME level increased and as the CP level decreased (p = 0.027).

### Fecal microbiota

**Figure 2:**
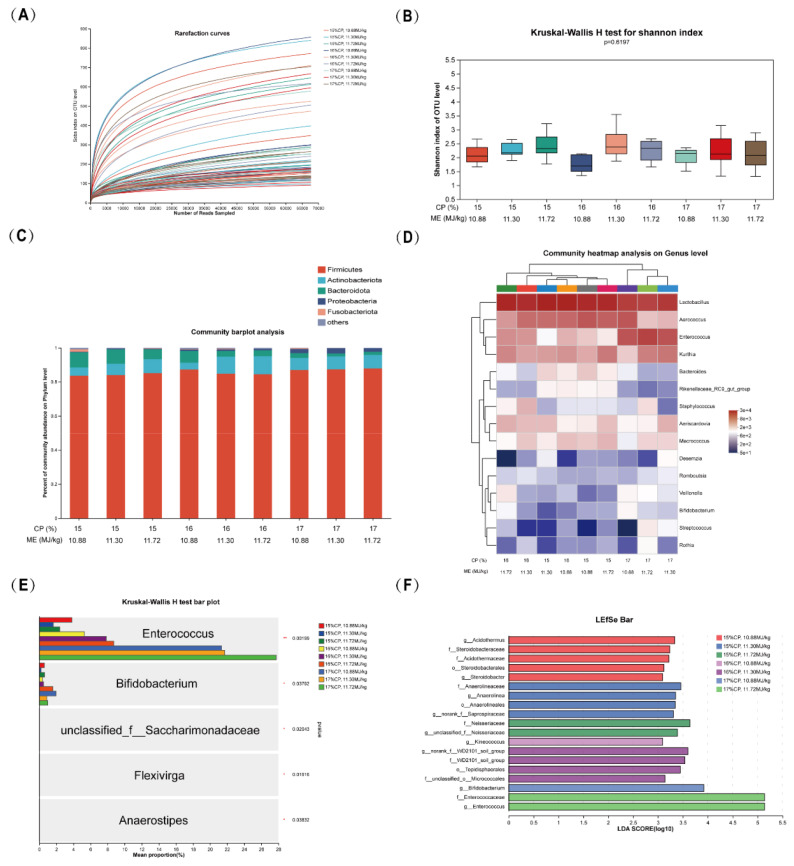
Effect of dietary ME and CP levels on fecal microbiota during the peak laying period of TSF. (A) Alpha index rarefraction curves graph. (B) Comparison of Shannon index between groups boxplot. (C) Relative abundance barplot at phylum level. (D) The community heatmap of top 15 at the genus level. (E) Top 5 significant different genus in dietary ME and CP levels. (F) The LEfSe analysis, with a LDA score above 3.0. ME, metabolizable energy; CP, crude protein; TSF, Taihe Silky Fowl; LEfSe, linear discriminant analysis effect size; LDA, linear discriminant analysis.

**Figure 3:**
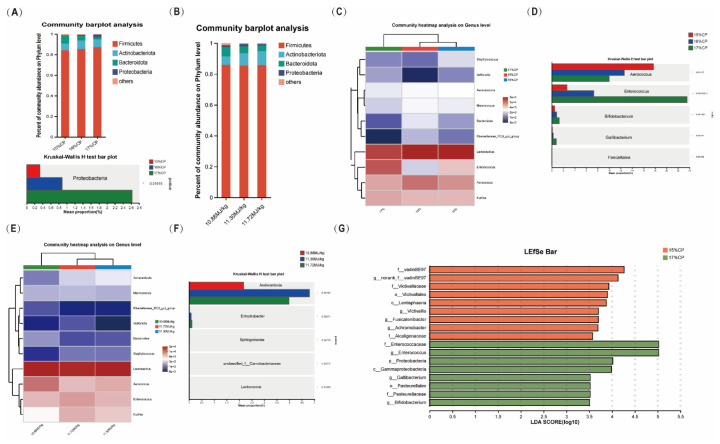
The main effect of dietary ME or CP levels on fecal microbiota during the peak laying period of TSF. (A) Relative abundance barplot at phylum level of three CP level. (B) Relative abundance barplot at phylum level of three ME level. (C) The community heatmap of top 10 at the genus level of three CP level. (D) Top 5 significant different genus of three CP level. (E) The community heatmap of top 10 at the genus level of three ME level. (F) Top 5 significant different genus of three ME level. (G) The LEfSe analysis, with a LDA score above 3.5. ME, metabolizable energy; CP, crude protein; TSF, Taihe Silky Fowl; LEfSe, linear discriminant analysis effect size; LDA, linear discriminant analysis.

In Figure 2A, all rarefaction curves are flattened, which indicates that the sequencing depth was sufficient for analyzing the true state of the bacterial community. In terms of alpha diversity, measured by the Shannon index, there were no significant differences between the 9 treatments (Figure 2B). At the phylum level, Firmicutes, Actinobacteriota, Bacteroidetes, and Proteobacteria dominated the fecal microbiota of TSF (Figures 2C, 3A, 3B), among which Firmicutes accounted for more than 80%. As shown in Figure 3A, with the CP level increasing, the abundance of Proteobacteria enhanced (p<0.05). We further displayed the top 15 (Figure 2D) or 10 (Figures 3C, 3E) heatmaps of community composition at the genus level and found that the top 10 dominant fecal microbiota genus of TSF were *Lactobacillus*, *Aerococcus*, *Enterococcus*, *Kurthia*, *Aeriscardovia*, *Macrococcus*, *Bacteroides*, *Staphylococcus*, *Rikenellaceae_RC9_gut_group*, and *Veillonella*. As shown in Figure 2E, *Enterococcus* and *Bifidobacterium* showed significant differences between the treatment groups. From the CP perspective, *Aerococcus*, *Enterococcus*, *Bifidobacterium* and *Gallibacterium* showed significant differences (Figure 3D). From the ME perspective, the genus was *Aeriscardovia*, and *Enhydrobacter* (Figure 3F). We extended the LDA score (LDA>3.0) histogram to uncover the key microbiota in each group based on the statistical significance, and we found that g__*Acidothermus*, g__*Steroidobacter*, g__*Anaerolinea*, g__*norank_f__Saprospiraceae*, g__*unclassified_f__Neisseriaceae*, g__*Kineococcus*, g__*norank_f__WD2101_soil_group*, g__*Bifidobacterium* and g__*Enterococcus* contributed the most to the between group differences (Figure 2F). From the CP perspective (Figure 3G), g__*norank_f__vadinBE97*, g__*Victivallis*, g__*Fusicatenibacter*, and g__*Achromobacter* were more abundant in the 15% CP group, g__*Enterococcus*, g__*Gallibacterium* and g__*Bifidobacterium* were more abundant in the 17% CP group, while no difference was found in the ME levels.

## DISCUSSION

Dietary ME and CP are two vital variables that affect the laying performance of hens and make up about 85% of the total diet cost [8]. To achieve the best growth performance, produce high-quality products and avoid feed waste, precision animal nutrition can provide animals with feed that precisely matches their nutritional requirements [9]. Animals instinctively feed for energy intake, and with the energy level increasing, the amount of feed intake gradually decreases [10,11]. Previous studies found that the ME and CP levels had an interaction effect on EW: the EW increased with the rising ME and CP level [12,13]. However, our study showed that there was no significant interaction effect of ME and CP levels on EW, but EW increased with CP in a concentration-dependent manner, consistent with a previous study [14]. Gunawardana et al [15] found that the increased CP level enhanced EP in Hy-Line W36 hens during 98 weeks to 110 weeks. Our results showed that the increased CP level improved EP was similar to the above findings. The reason might be that hens had a high need for nutrients during the peak laying period, especially protein, increasing the dietary CP level helped them to ingest sufficient CP for EP. A previous study indicated that CP levels (15%, 16.5%, and 18%) affected the EM of White Leghorn layers; as the CP level increased, the EM increased significantly in 21 to 32 weeks and had an increasing trend in 33 to 52 weeks [16]. Other studies reported that the ME or CP level can reduce the FCR [13,17]. These findings are consistent with our results. It can be seen that different scholars came to varying conclusions, possibly because of be differences in breed, breeding conditions, diet sources, and different levels of ME or CP. Especially in terms of breed, TSF is a local breed in China, with a low EP and a shorter peak laying period compared to commercial laying hens. Thus, increasing the CP level to an appropriate level is beneficial for improving EP during the peak laying period.

It has been reported that dietary ME or CP levels can affect egg quality to some extent. In the assessment of the egg’s internal characteristics, albumen height and Haugh unit are important indicators to measure egg albumen quality and freshness [18]. Previous research has shown that the dietary ME or CP level affected the albumen height and Haugh unit in Beijing You Chicken at 30 weeks [19]. In contrast, Ding et al [20] found that albumen height and Haugh unit was not affected by dietary ME or CP level for Feng-da-1 layers. With the dietary ME level increasing from 2,550 kcal/kg to 2,650 kcal/kg, the yolk color tended to increase [21]. In terms of CP, Wu et al [8] reported that the yolk color linearly decreased as the CP levels increased from 14.9% to 16.1% of Hy-Line W36 hens during 40 weeks to 52 weeks. Kang et al [22] demonstrated that eggshell thickness and eggshell strength were not affected by differences in ME levels. There were no significant main or interaction effects between ME and CP levels in influencing egg quality [13], which was consistent with our study. There might be several reasons for the above difference. First, the breeds, ages and diets were different. Secondly, there was a relatively large individual variation in the eggs of TSF because they were not systematically selected and bred. Going forward, the detection of more egg quality samples should make the sample statistics more robust.

Physiological, nutritional, and pathological changes in animals could be reflected in certain serum indicators. For example, serum TG and T-CHO concentrations could reflect the absorption and metabolism of lipids. In contrast to LDL-C, HDL-C promotes the uptake of cholesterols from peripheral tissues and transport to the liver for catabolism [23]. We found that with the ME level increasing, the concentrations of TG, T-CHO and HDL rose significantly, while the LDL-C decreased significantly in serum. A previous study found that fat birds had higher HDL-C, and lower LDL-C levels than lean birds [24], suggesting that high-energy diets may cause obesity tendency. The apparent digestibility can reflect the digestion and absorption of diet nutrients to some extent, likewise, the level of nutrient utilization in hens also directly affects their laying performance. Previous studies on the effect of ME and CP levels on the apparent digestibility of nutrients often focused on pigs, ruminants and broilers, while few studies have been conducted on laying hens. Cui [25] found that as the CP level decreased, the digestibility of CP of broiler tended to increase, which is in agreement with our study. In addition, with the ME level increasing, the apparent digestibility of gross energy linearly also increased [22], consistent with our results. The reason might be that we added soybean oil to the high-energy feed (11.72 MJ/kg), which extended the digestive transit time for the feed, such that it could be more fully exposed to digestive enzymes, ultimately improving nutrient utilization [26].

In recent years, the intestinal microbiota has been shown to play an important role in the immune regulation, nutrient digestion, growth performance, and physical health of poultry [27]. Intestinal microbiota is influenced by genotype, sex, age, diet, feeding environment and so on. A previous study had found that the intestinal microbiota of broilers is influenced by dietary factors during feeding and that the composition of the diet has a greater impact on the intrinsic intestinal microbiota than environmental factors [28]. Chen [29] discovered that different ME level changed the composition of gut microbiota in Jingfen No.2 layer breeders during the late laying period: low ME diets increased the abundance of *Parasutterella* and *Sutterella* in the cecum, while it reduced the abundance of *Romboutsia*. There is a lack of research on the intestinal microbiota of TSF, which necessitates exploring the changes in diet ingredients and how to change the intestinal microbiota, especially ME and CP levels. Owing to the convenience and non-invasiveness of fecal sampling, feces is commonly analyzed as representative of the intestinal microbiota [30]. The normal intestinal microbiota of laying hens is dominated by Firmicutes, Bacteroidetes, Proteobacteria, Fusobacteria, and Actinobacteria at the phylum level [31], in agreement with our results. Firmicutes and Bacteroidetes, are the two dominant phyla in poultry, which have a strong correlation with the host’s health status. As temporally the most unstable of intestinal microbiota, there are many opportunistic pathogens in Proteobacteria, including *Escherichia coli*, *Salmonella*, *Campylobacter*, and *Helicobacter* [32], which have been associated with obesity and disease [33]. A previous study found that the abundance of Proteobacteria was significantly increased with the dietary CP levels, compared with the 15% CP level, the abundance of Proteobacteria at 16% CP level increased by 107% [34]. At the genus level, Xiao et al [35] found that *Lactobacillus*, *Enterococcus*, *Bacteroides*, and *Corynebacterium* were the major microbial genus inhabiting 5 different intestinal locations in 42-day-old Ross 308 broiler chicks; specifically, *Lactobacillus*, *Enterococcus* were dominant in the rectum, similar to our results. To find the key microbial with significant differences, we analyzed the differences between groups and the LEfSe to clarify this issue. *Victivallis* is abundant in the digestive tract of roughage-tolerant animals such as cattle and sheep, capable of degrading fibers, and positively correlated with volatile fatty acid production [36,37]. Aside from *Victivallis* in the 15% CP group, we found a significantly higher abundance of *Enterococcus* and *Bifidobacterium* in the 17% CP group. *Enterococcus* is a lactic acid bacteria comprising both pathogenic and commensal microbiota ubiquitous in the environment, even as natural colonizers of the gastrointestinal tract of humans and most animals [38,39]. *Bifidobacterium* is an important probiotic in the intestine of humans and animals, playing an essential role in maintaining the balance of gut microbiota, inhibiting the invasion and colonization of pathogenic bacteria, and regulating the body’s immune system [40,41]. A previous study found that dietary supplementation of 0.1% *Bifidobacterium* animals improved laying performance and egg quality in Lohmann White hens [42]. *Gallibacterium* is a potential opportunistic pathogenic bacterium, and its subspecies *Gallibacterium anatis* could cause a decrease in EP and increase the risk of peritonitis in laying hens [43]. From this, we can infer that, based on the shorter peak laying period of TSF, although a high dietary CP diet can increase EP, excessive protein fermentation in the hindgut can cause an increase in pathogenic bacteria, which may harm the health of hens. Therefore, it is necessary to timely reduce the CP level in the diet after the peak laying period of TSF.

## CONCLUSION

Dietary ME and CP levels significantly affect the laying performance of TSF during the peak laying period. With the CP levels increasing, the EP, EW, and EM increased, while FCR decreased. As the ME levels increased, the ADFI and FCR decreased. Different levels of ME and CP changed the composition of fecal microbiota, 17% CP increased the abundance of *Bifidobacterium*. The present study suggested that 10.88 MJ/kg dietary ME and 17% CP level can be helpful for increasing the laying performance and benefit for gut microbiota of TSF during the peak laying period.

## Figures and Tables

**Table 1:** Composition and nutrient levels of the experimental diets (air-dried basis)

CP (%)	CP:15			CP:16			CP:17		
ME (MJ/kg)	10.88	11.30	11.72	10.88	11.30	11.72	10.88	11.30	11.72
ME (kcal/kg)	2,600	2,700	2,800	2,600	2,700	2,800	2,600	2,700	2,800

Ingredients (%)	100	100	100	100	100	100	100	100	100
Corn	31.56	35.16	32.76	28.26	31.86	29.46	24.96	28.56	26.06
Soybean meal 43%	21.30	20.60	21.00	22.20	21.50	21.90	23.10	22.40	22.90
Extruded soybean	0.60	0.60	0.60	3.00	3.00	3.00	5.40	5.40	5.40
Rice bran	8.00	8.00	8.00	8.00	8.00	8.00	8.00	8.00	8.00
Broken rice	25.00	25.00	25.00	25.00	25.00	25.00	25.00	25.00	25.00
Limestone meal	8.20	8.20	8.20	8.20	8.20	8.20	8.20	8.20	8.20
CaHPO_4_	1.30	1.30	1.30	1.30	1.30	1.30	1.30	1.30	1.30
NaCl	0.40	0.40	0.40	0.40	0.40	0.40	0.40	0.40	0.40
Multi-vitamin^1)^	0.25	0.25	0.25	0.25	0.25	0.25	0.25	0.25	0.25
Mineral premix^2)^	0.25	0.25	0.25	0.25	0.25	0.25	0.25	0.25	0.25
Choline chloride	0.10	0.10	0.10	0.10	0.10	0.10	0.10	0.10	0.10
Zeolite powder	2.90	0	0	2.90	0	0	2.90	0	0
Soybean oil	0	0	2.00	0	0	2.00	0	0	2.00
DL-Methionine	0.125	0.125	0.125	0.125	0.125	0.125	0.125	0.125	0.125
Compound enzyme^3)^	0.015	0.015	0.015	0.015	0.015	0.015	0.015	0.015	0.015
Nutrient level									
ME (MJ/kg) (calculated)	10.89	11.31	11.72	10.88	11.30	11.72	10.88	11.29	11.71
ME (kcal/kg) (calculated)	2,602.33	2,701.57	2,801.25	2,600.94	2,700.18	2,799.85	2,599.54	2,698.78	2,797.59
CP (%) (calculated)	15.02	15.01	14.99	15.99	15.98	15.96	16.96	16.95	16.96
CP (%) (analyzed)	14.99	15.00	14.97	15.97	15.96	15.95	16.94	16.95	16.94
Ca (%) (analyzed)	3.48	3.50	3.49	3.51	3.52	3.46	3.53	3.49	3.50
Total phosphorus (%) (analyzed)	0.42	0.45	0.41	0.41	0.43	0.43	0.40	0.41	0.43
Lysine (%) (analyzed)	0.82	0.79	0.81	0.86	0.84	0.88	0.92	0.95	0.93
Methionine (%) (analyzed)	0.37	0.39	0.36	0.37	0.38	0.39	0.37	0.39	0.36
Methionine+cystine (%) (analyzed)	0.64	0.66	0.63	0.65	0.68	0.66	0.65	0.64	0.66

CP, crude protein; ME, metabolizable energy.

1) Multi-vitamin provided the following per kg of diet: vitamin A, 12,000 IU; vitamin E, 49.5 mg; vitamin B_1_, 3 mg; vitamin B_2_, 10.5 mg; vitamin B_6_, 6 mg; vitamin B_12_, 0.03 mg; vitamin D_3_, 3750 IU; vitamin K_3_, 6 mg; Folic acid, 3 mg; nicotinic acid, 60 mg; pantothenic acid, 18 mg; biotin, 0.3 mg.

2) Mineral premix provided the following per kg of diet: Mn, 60 mg; Zn, 120 mg; Cu, 10 mg; Fe, 80 mg; I, 1.1 mg; Se, 0.4 mg; Co, 0.2 mg.

3) Compound enzyme: Xylanase≥22,000 U/g; β-glucan≥3,000 U/g; β-mannanase≥350 U/g; cellulase≥300 U/g; amylase≥3,200 U/g; protease≥2,500 U/g.

**Table 2:** Effect of dietary ME and CP levels on laying performance during the peak laying period of TSF

CP (%)	ME (MJ/kg)	ADFI (g/d)	EW (g)	EP (%)	EM (g/d)	FCR (g/g)
15	10.88	60.32^a^	36.37^d^	54.91^c^	19.98^bc^	3.02^a^
	11.30	59.53^ab^	36.20^d^	54.84^c^	19.84^c^	3.00^a^
	11.72	57.57^bc^	36.67^c^	55.27^bc^	20.26^abc^	2.84^abc^
16	10.88	59.61^a^	36.33^d^	55.79^abc^	20.15^bc^	2.96^ab^
	11.30	58.30^abc^	36.25^d^	55.60^abc^	20.09^bc^	2.90^ab^
	11.72	57.03^c^	36.33^d^	55.67^abc^	20.12^bc^	2.83^abc^
17	10.88	59.81^a^	36.94^c^	57.52^ab^	21.24^ab^	2.82^abc^
	11.30	58.28^abc^	37.80^a^	56.01^abc^	21.17^abc^	2.75^bc^
	11.72	56.98^c^	37.38^b^	57.74^a^	21.56^a^	2.64^c^
	SEM	0.22	0.03	0.23	0.13	0.02
Main effect						
CP	15	59.14	36.41^b^	55.01^b^	20.03^b^	2.96^a^
	16	58.31	36.30^b^	55.69^ab^	20.12^b^	2.89^b^
	17	58.36	37.37^a^	57.09^a^	21.32^a^	2.74^b^
	p-value	0.224	0.023	0.047	0.022	0.023
ME	10.88	59.91^a^	36.54	56.07	20.46	2.93^a^
	11.30	58.70^b^	36.75	55.78	20.37	2.89^ab^
	11.72	57.20^c^	36.79	56.23	20.65	2.77^b^
	p-value	<0.001	0.074	0.367	0.654	0.045
Interaction effect						
CP×ME	p-value	0.475	0.078	0.710	0.681	0.769

Data are represented as mean & SEM (n = 5).

ME, metabolizable energy; CP, crude protein; TSF, Taihe Silky Fowl; ADFI, average daily feed intake; EW, egg weight; EP, egg production; EM, egg mass; FCR, feed conversion ratio; SEM, standard error of the mean.

EM = EP×EW, FCR = ADFI/EM.

a–d Within a column, means with different superscripts are significantly different p<0.05.

**Table 3:** Effect of dietary ME and CP levels on egg quality during the peak laying period of TSF

CP (%)	ME (MJ/kg)	Albumen height (mm)	Haugh unit	Yolk color	Eggshell weight (g)	Eggshell thickness (mm)	Eggshell strength (kgf/m^2^)
15	10.88	4.28	71.93	3.90	4.36	0.31	4.01
	11.30	4.10	72.97	4.10	4.36	0.31	4.11
	11.72	4.48	74.03	4.10	4.63	0.32	4.10
16	10.88	4.19	73.45	4.00	4.57	0.31	3.92
	11.30	4.11	71.09	3.80	4.52	0.32	3.97
	11.72	4.52	74.13	3.90	4.55	0.31	3.94
17	10.88	4.15	70.76	4.13	4.49	0.31	4.16
	11.30	4.20	72.00	4.00	4.71	0.31	4.16
	11.72	4.36	72.44	3.90	4.71	0.32	4.05
	SEM	0.07	0.67	0.08	0.03	0.00	0.06
Main effect							
CP	15	4.28	73.02	4.03	4.41	0.31	4.07
	16	4.25	72.78	3.90	4.54	0.31	3.94
	17	4.23	71.61	4.00	4.61	0.31	4.12
	p-value	0.962	0.725	0.800	0.105	0.613	0.453
ME	10.88	4.19	71.95	4.00	4.47	0.31	4.02
	11.30	4.13	72.10	3.97	4.52	0.31	4.08
	11.72	4.45	73.53	3.98	4.64	0.31	4.03
	p-value	0.216	0.625	0.976	0.234	0.124	0.930
Interaction effect							
CP×ME	p-value	0.978	0.910	0.909	0.402	0.058	0.988

Data are represented as mean & SEM (n = 30).

ME, metabolizable energy; CP, crude protein; TSF, Taihe Silky Fowl; SEM, standard error of the mean.

**Table 4:** Effect of dietary ME and CP levels on serum routine biochemical indicators during the peak laying period of TSF

CP (%)	ME (MJ/kg)	ALT (U/L)	AST (U/L)	TP (g/L)	ALB (g/L)	GLOB (g/L)	A/G
15	10.88	3.52	201.25	49.49	16.15	33.34	0.49
	11.30	3.49	203.57	50.55	16.23	34.33	0.48
	11.72	3.54	201.00	50.92	16.44	34.46	0.48
16	10.88	3.60	205.29	52.09	16.45	35.64	0.47
	11.30	3.74	203.80	52.01	16.38	35.64	0.47
	11.72	3.64	191.14	53.00	16.91	36.09	0.48
17	10.88	3.44	191.63	53.19	16.57	36.61	0.47
	11.30	3.55	202.80	52.40	16.67	35.73	0.48
	11.72	3.47	195.33	54.95	16.80	38.15	0.45
	SEM	0.05	2.64	0.65	0.14	0.63	0.01
Main effect							
CP	15	3.52	202.00	50.31	16.27	34.04	0.48
	16	3.66	199.68	52.37	16.58	35.79	0.48
	17	3.48	195.74	53.58	16.69	36.89	0.47
	p-value	0.288	0.736	0.090	0.500	0.097	0.492
ME	10.88	3.52	198.68	51.52	16.38	35.13	0.48
	11.30	3.59	203.41	51.62	16.41	35.21	0.48
	11.72	3.55	196.09	52.95	16.72	36.89	0.47
	p-value	0.805	0.545	0.634	0.608	0.759	0.955
Interaction effect							
CP×ME	p-value	0.974	0.790	0.880	0.993	0.871	0.857

Data are represented as mean & SEM (n = 10).

ME, metabolizable energy; CP, crude protein; TSF, Taihe Silky Fowl; ALT, alanine aminotransferase; AST, aspartate aminotransferase; TP, total protein; ALB, albumin; GLOB, globulin; A/G, albumin/globulin; SEM, standard error of the mean.

**Table 5:** Effect of dietary ME and CP levels on serum lipid metabolism indices during the peak laying period of TSF

CP (%)	ME (MJ/kg)	TG (mmol/L)	TCHO (mmol/L)	LDL-C (mmol/L)	HDL-C (mmol/L)	UA (μmol/L)
15	10.88	3.98^ab^	2.77^ab^	1.37^a^	1.14^b^	222.00
	11.30	4.27^ab^	2.95^ab^	1.16^ab^	1.33^ab^	227.88
	11.72	4.47^ab^	3.29^ab^	1.02^ab^	1.43^ab^	235.40
16	10.88	4.08^ab^	2.70^b^	1.32^a^	1.15^ab^	247.43
	11.30	4.31^ab^	3.08^ab^	1.19^ab^	1.29^ab^	248.57
	11.72	4.52^a^	3.30^ab^	1.04^ab^	1.46^a^	252.38
17	10.88	3.96^b^	2.75^ab^	1.31^ab^	1.19^ab^	261.14
	11.30	4.31^ab^	2.90^ab^	1.12^ab^	1.31^ab^	267.71
	11.72	4.50^ab^	3.36^a^	0.94^b^	1.47^a^	263.00
	SEM	0.05	0.06	0.03	0.04	6.06
Main effect						
CP	15	4.27	2.99	1.19	1.30	227.70
	16	4.29	3.04	1.18	1.32	249.59
	17	4.25	3.00	1.13	1.33	264.05
	p-value	0.678	0.578	0.850	0.760	0.083
ME	10.88	4.01^b^	2.74^b^	1.33^a^	1.16^b^	243.52
	11.30	4.30^ab^	2.97^ab^	1.15^ab^	1.31^ab^	247.14
	11.72	4.49^a^	3.31^a^	1.01^b^	1.46^a^	250.61
	p-value	0.037	0.041	0.039	0.028	0.906
Interaction effect						
CP×ME	p-value	0.688	0.451	0.755	0.882	0.998

Data are represented as mean & SEM (n = 10).

ME, metabolizable energy; CP, crude protein; TSF, Taihe Silky Fowl; TG, triglycerides; TCHO, total cholesterol; LDL-C, low-density lipoprotein cholesterol; HDL-C, high-density lipoprotein cholesterol; UA, uric acid; SEM, standard error of the mean.

a,b Within a column, means with different superscripts are significantly different p<0.05.

**Table 6:** Effect of dietary ME and CP levels on the apparent digestibility of nutrients during the peak laying period of TSF

CP (%)	ME (MJ/kg)	Dry matter (%)	Crude protein (%)	Gross energy (%)
15	10.88	76.52	72.86^abc^	77.86^c^
	11.30	77.11	73.35^ab^	78.34^c^
	11.72	76.64	73.72^a^	80.83^ab^
16	10.88	76.42	72.22^bc^	78.25^c^
	11.30	75.50	73.31^ab^	79.06^bc^
	11.72	76.48	73.39^ab^	79.40^bc^
17	10.88	75.95	72.00^c^	77.25^c^
	11.30	75.67	72.57^abc^	78.00^c^
	11.72	76.83	72.32^bc^	81.93^a^
	SEM	0.15	0.14	0.33
Main effect				
CP	15	76.76	73.31^a^	79.01
	16	76.13	72.97^ab^	78.90
	17	76.15	72.30^b^	79.06
	p-value	0.119	0.027	0.654
ME	10.88	76.30	72.36^b^	77.78^b^
	11.30	76.09	73.08^ab^	78.47^b^
	11.72	76.65	73.14^a^	80.72^a^
	p-value	0.249	0.029	0.018
Interaction Effect				
CP×ME	p-value	0.202	0.738	0.166

Data are represented as mean & SEM (n = 10).

ME, metabolizable energy; CP, crude protein; TSF, Taihe Silky Fowl; SEM, standard error of the mean.

a–c Within a column, means with different superscripts are significantly different p<0.05.
